# Cell Density-dependent Anammox Activity of *Candidatus* Brocadia sinica Regulated by *N*-acyl Homoserine Lactone-mediated Quorum Sensing

**DOI:** 10.1264/jsme2.ME20086

**Published:** 2020-10-24

**Authors:** Mamoru Oshiki, Haruna Hiraizumi, Hisashi Satoh, Satoshi Okabe

**Affiliations:** 1 Division of Environmental Engineering, Faculty of Engineering, Hokkaido University, North-13, West-8, Sapporo, Hokkaido 060–8628, Japan

**Keywords:** anaerobic ammonium oxidation (anammox), *Ca.* Brocadia sinica, cell density-dependent regulation, *N*-acyl homoserine lactone, quorum sensing

## Abstract

The activity of anaerobic ammonia-oxidizing (anammox) bacteria is considered to depend on cell density; however, this has not yet been confirmed due to the fastidious nature of anammox bacteria (*e.g.*, slow growth, oxygen sensitivity, and rigid aggregate formation). In the present study, the cell density-dependent occurrence of anammox activity (^14-15^N_2_ gas production rate) was investigated using planktonic enrichment cultures of *Candidatus* Brocadia sinica. This activity was detectable when the density of cells was higher than 10^7^‍ ‍cells‍ ‍mL^–1^ and became stronger with increases in cell density. At the cell densities, the transcription of the BROSI_A1042 and BROSI_A3652 genes, which are potentially involved in the biosynthesis and reception of *N*-acyl homoserine lactone (AHL), was detectable in *Brocadia sinica* cells. The presence of AHL molecules in the MBR culture of *B. sinica* was confirmed by an AHL reporter assay and gas chromatography mass spectrometry analysis. The exogenous addition of the MBR culture extract and AHL molecules (a cocktail of C_6_, C_8_, C_10_, and C_12_-homoserine lactones) increased the specific ^14-15^N_2_ production rate of *B. sinica*. These results suggest that the specific anammox activity of *B. sinica* is regulated by AHL-mediated quorum sensing.

Anaerobic ammonium oxidation (anammox) is a microbial process in which NH_4_^+^ is oxidized to N_2_ gas using NO_2_^–^ as an electron accepter (Mulder *et al.*, 1995). Anammox activity has been detected from a wide range of anoxic environments and significantly contributes to fixed nitrogen loss (Oshiki *et al.*, 2016). The anammox process is mediated by a monophyletically group, which is affiliated with the bacterial order *Brocadiales*. To date, the following five candidate genera have been proposed: *Kuenenia*, *Brocadia*, *Jettenia*, *Anammoxoglobus*, and *Scalindua* (Jetten *et al.*, 2009). The distributions of anammox bacterial 16S rRNA gene sequences in natural and man-made environments indicate that each anammox genus appears to have a defined niche (Sonthiphand *et al.*, 2014). For example, *Brocadia sinica* has frequently been detected in NH_4_^+^- and NO_2_^–^-rich environments, such as wastewater treatment plants (Zhang *et al.*, 2017a).

We successfully obtained enrichment cultures of *B. sinica*, *Brocadia sapporoensis*, *Jettenia caeni*, and *Scalindua japonica* from these 5 genera (Tsushima *et al.*, 2007; Kindaichi *et al.*, 2011; Hira *et al.*, 2012; Narita *et al.*, 2017). Sudden marked increases in anammox activity are frequently observed during enrichment culturing processes (Tsushima *et al.*, 2007), and cannot be simply explained by Monod growth kinetics. Furthermore, these anammox bacteria may form dense microbial aggregates and biofilms (Ali *et al.*, 2018), which exhibit strong anammox activity. These findings suggest that anammox activity is regulated in a cell density-dependent manner. Previous studies indicated the occurrence of cell density-dependent anammox activity. *Brocadia anammoxidans*, *Kuenenia stuttgartiensis*, and anammox biofilms required minimum cell densities of >10^10^‍ ‍cells‍ ‍mL^–1^ (Strous *et al.*, 1999), 2×10^8^‍ ‍cells‍ ‍mL^–1^ (Egli *et al.*, 2001), and 3.1‍ ‍g volatile suspended solids (VSS) L^–1^ (Clippeleir *et al.*, 2011), respectively, to exhibit anammox activity. These minimum cell densities were confirmed using dispersed biofilms and granules, not planktonic free-living cells.

Difficulties are associated with the dispersal of anammox biofilms and granules using conventional homogenization techniques, by which only fragmented biofilms and granules are generated (Oshiki *et al.*, 2011). Thus, local cell densities in fragmented biofilms and granules remain very high and cannot be accurately quantified. Furthermore, broken fragments display strong heterogeneity with respect to both the microorganisms present and their physicochemical microenvironments due to substrate diffusion limitations (Okabe *et al.*, 1999a; 1999b; Ito *et al.*, 2002; Kindaichi *et al.*, 2007; Satoh *et al.*, 2007). Furthermore, anammox activity has been erroneously evaluated as the rate of NH_4_^+^ and/or NO_2_^–^ consumption (Strous *et al.*, 1999; Egli *et al.*, 2001; Clippeleir *et al.*, 2011) due to the presence of other coexisting microorganisms, such as nitrifiers and denitrifiers (Kindaichi *et al.*, 2012; Lawson *et al.*, 2017). Due to these experimental limitations, the cell density-dependent regulation of anammox activity remains unresolved. A continuous cultivation method of planktonic *B. sinica* cells using a membrane bioreactor (MBR) (Oshiki *et al.*, 2013; Zhang and Okabe, 2017b) and a ^15^NH_4_^+^ tracer technique in which the production of ^14-15^N_2_ gas (a specific marker of anammox activity) is quantified by gas chromatography mass spectrometry (GC/MS) (Ali *et al.*, 2015) has been established. These experimental techniques provide an excellent opportunity to examine the cell density-dependent anammox activity of *B. sinica*.

*N*-acyl homoserine lactone (AHL) is a well-known signaling molecule involved in cell-cell communication and cell density-dependent behavior and is synthesized by a wide range of Gram-negative bacteria (Parsek and Greenberg, 2000). AHL molecules have been identified in enrichment cultures of anammox bacteria, and the addition of AHL molecules has been shown to enhance anammox activity (Tang *et al.*, 2015; Sun *et al.*, 2018; Tang *et al.*, 2018). Furthermore, candidate genes (*i.e.*, JqsI-1 and JqsI-2) involved in the biosynthesis of AHL molecules have been identified in the *J. caeni* genome, and the JqsI-1 and JqsI-2 proteins expressed in a heterologous manner in *Escherichia coli* cells synthesized AHL molecules (Tang *et al.*, 2019). The presence of AHL molecules induced the transcription of *hzsA* mRNA encoding hydrazine synthase; however, a specific gene(s) involved in the reception of AHL molecules and subsequent metabolic regulation in anammox bacterial cells remains missing (Tang *et al.*, 2019). To date, only one study has described the specific anammox bacterial genes involved in the biosynthesis and reception of AHL molecules. Limited information is currently available on AHL-mediated quorum sensing (QS) in the anammox process, and, thus, further studies are needed on other anammox bacterial species.

The aims of the present study were as follows: 1) to examine cell density-dependent specific anammox activity using a highly enriched planktonic cell culture of *B. sinica* and the ^15^NH_4_^+^ tracer technique, 2) to identify the genes involved in the biosynthesis and reception of AHL molecules by *B. sinica*, and 3) to confirm the presence of AHL molecules in a culture of *B. sinica*, which regulates specific anammox activity. In the present study, specific anammox activity was carefully assessed as the ^14-15^N_2_ production rate per cell using a serially diluted culture of planktonic *B. sinica* cells (10^6^–10^10^ cell mL^–1^). The influence of the external addition of AHL molecules (a cocktail of C_6_, C_8_, C_10_, and C_12_-homoserine lactones) on the specific ^14-15^N_2_ gas production rate of *B. sinica* was examined in more detail. The results obtained demonstrated the cell density-dependent anammox activity of *B. sinica*, which appears to be regulated by AHL-mediated QS.

## Materials and Methods

### *B. sinica* biomass

The planktonic and granular biomasses of *B. sinica* cells were subjected to the following activity assay. The planktonic biomass was cultivated in a MBR as previously described (Oshiki *et al.*, 2013). Briefly, a 3-L jar fermenter (MBF-500ME; EYELA) equipped with a hollow fiber membrane unit (300 polyethylene tubes, pore size 0.1‍ ‍μm, tube diameter 1‍ ‍mm, and length 70‍ ‍mm) was operated at 37°C in the dark. An inorganic medium containing 40‍ ‍mM NH_4_(SO_4_)_2_, 40‍ ‍mM NaNO_2_, 0.18‍ ‍mM KH_2_PO_4_, 0.243‍ ‍mM MgSO_4_ 7H_2_O, 0.46‍ ‍mM CaCl_2_, 1‍ ‍mg L^–1^ yeast extract (Becton, Dickinson and Company), and 0.5‍ ‍mL L^–1^ trace element solution I and II (van de Graaf *et al.*, 1996) was supplied into the MBR at a hydraulic retention time (HRT) of 2 days, resulting in a nitrogen loading rate of 0.3 kg-N m^–3^ d^–1^. The inside of the MBR was continuously mixed with a metal propeller at 160 rpm and sparged with 95%Ar-5%CO_2_ at a flow rate of 10‍ ‍mL min^–1^. The pH of the culture was not controlled, but ranged between pH 7.6 and 8.6. The MBR was operated for more than 1 year with a biomass retention time of 30 days, and the cell density of the culture was maintained at approximately 10^10^‍ ‍cells‍ ‍mL^–1^.

The granular biomass was cultivated in a 60-mL up-flow column reactor operated at 37°C in the dark as previously described (Tsushima *et al.*, 2007). An inorganic medium containing 10‍ ‍mM (NH_4_)_2_SO_4_, 10‍ ‍mM NaNO_2_, 1.22‍ ‍mM MgSO_4_·7H_2_O, 1.22‍ ‍mM CaCl_2_ 2H_2_O, 0.2‍ ‍mM KH_2_PO_4_, 5‍ ‍mM KHCO_3_, and 0.5‍ ‍mL L^–1^ trace element solution I and II (van de Graaf *et al.*, 1996) was continuously supplied with HRT of 0.25 h^–1^, resulting in a nitrogen loading rate of 41 kg-N m^–3^ d^–1^. This nitrogen loading rate was an order of magnitude greater than that of the above MBR, while the biomass-specific nitrogen loading rate was similar between the reactors because the biomass concentration in the column reactor was also one order of magnitude greater (*i.e.*, 10–20 and 0.5–1.5‍ ‍g dry weight biomass L^–1^ in the column reactor and MBR, respectively). The column reactor was continuously operated for more than 1 year, and the effluent NO_2_^–^ concentration was maintained at less than 0.1‍ ‍mM. The granular biomass with an average diameter of 2.5‍ ‍mm was collected from the reactor using a spatula and homogenized well with a glass tissue homogenizer (Thomas Scientific). The biomass was grinded manually in a glass pot using a glass pestle.

The dominance of *B. sinica* cells in the above planktonic and granular biomasses (>90% of the total biomass) was assessed by measuring the abundance of *B. sinica* in the total biomass using fluorescence *in situ* hybridization (FISH) with the AMX820 oligonucleotide probe as previously described (Kindaichi *et al.*, 2007).

### Activity test

An anoxic batch incubation using ^15^NH_4_^+^-labeled substrate was performed as previously described (Ali *et al.*, 2015). Standard anaerobic techniques were used with an anaerobic chamber (Coy Laboratory Products). Briefly, the planktonic and dispersed granular biomasses were washed twice and serially diluted using the inorganic medium without NH_4_^+^ and NO_2_^–^ to a cell density of 10^6^–10^10^‍ ‍cells‍ ‍mL^–1^. Ten milliliters of the cell suspension was dispensed into 25-mL glass vials (Nichidenrika), which were sealed with a butyl rubber stopper and aluminum seals. After replacing the headspace with helium gas, anoxic ^15^NH_4_^+^ (Cambridge Isotope Laboratories) and ^14^NO_2_^–^ stock solutions were dispensed to a final concentration of 2.5‍ ‍mM, and the vials were incubated at 37°C in the dark for up to 340 h. The incubation was performed in triplicate and with negative control vials (without *B. sinica* cells) to examine abiotic gas production. Penicillin G, an inhibitor of heterotrophic denitrifier, was supplemented into the vials at a final concentration of 500‍ ‍μg mL^–1^.

The influence of the AHL cocktail and ethyl acetate extract of the culture supernatant was examined using planktonic *B. sinica* cells. The cell suspension was diluted to 7.2×10^7^‍ ‍cells‍ ‍mL^–1^, and 10‍ ‍mL of the cell suspension was dispensed into 25-mL glass vials as described above. Ten microliters of the ethyl acetate extract (see below) and AHL cocktail was dispensed into the vials, which were incubated as described above. The AHL cocktail contained C_6_-HSL, C_8_-HSL, C_10_-HSL, and C_12_-HSL (100‍ ‍μM each), which were purchased from the University of Nottingham.

### Chemical analysis

The concentration of ^14-15^N_2_ gas was assessed by GC/MS as previously described (Isobe *et al.*, 2011). Ten microliters of headspace gas was injected into a GCMS-QP2010SE gas chromatograph (Shimadzu) equipped with a CP-Pora BOND Q fused silica capillary column (Agilent Technologies). A standard curve was prepared using diluted ^15-15^N_2_ gas (Cambridge Isotope Laboratories).

Cell density was assessed by measuring the total protein concentration. Total protein was extracted using 10% SDS solution as previously described (Oshiki *et al.*, 2011), and protein concentrations were measured using a DC protein assay kit (Bio-Rad) according to the manufacturer’s instructions. Bovine serum albumin was used as a protein standard. Cell density was calculated from the total protein concentration using a conversion factor of 4×10^10^ cells mg protein^–1^ (the present study). Cell numbers were measured using a direct cell counting method (Oshiki *et al.*, 2020). Briefly, cells were collected on a black polycarbonate membrane filter (pore size; 0.2‍ ‍μm) (Advantec), stained with 2‍ ‍μg mL^–1^ 4′,6-diamidino-2-phenylindole (DAPI) solution, and the number of cells was manually counted under a fluorescence microscope.

The detection and identification of AHLs in the *B. sinica* culture were performed by GC/MS and AHL reporter assays. The extraction and concentration of AHL molecules from the *B. sinica* culture were performed as previously described (Shaw *et al.*, 1997). One liter of reactor effluents (effluent of the up-flow column reactor and membrane filtrate of the MBR) was centrifuged at 8,000×*g* for 20‍ ‍min to obtain a cell-free supernatant. The supernatant was collected, and pH was adjusted to pH 2 by the addition of 1N HCl. After 12 h of incubation at 20°C, the supernatant was extracted twice using equal volumes of ethyl acetate, and ethyl acetate extracts were pooled. Extracts were concentrated using a rotary evaporator to a final volume of 200‍ ‍μL. The AHL cocktail was prepared by dissolving C_6_-HSL, C_8_-HSL, C_10_-HSL, and C_12_-HSL chemicals at a final concentration of 100‍ ‍μM in ethyl acetate.

The AHL reporter assay using *Chromobacterium violaceum* strains CV026 and VIR24 was performed as previously described (McClean *et al.*, 1997; Someya *et al.*, 2009). These bacteria produce violacein when the culture contains submillimolar concentrations of short- and long-acyl-chain compounds (C_4_ to C_7_ and C_6_ to C_14_ AHLs, respectively). Both bacterial strains were aerobically cultivated at 30°C using Luria-Bertani medium. One milliliter of the culture (early stationary phase) was mixed with 50‍ ‍mL of 1%-agar LB medium containing 50‍ ‍mM MOPS (pH 7.0), which was subsequently solidified in a Petri dish. Sterile cellulose paper disks (diameter of 5‍ ‍mm) (Advantec) were placed on the agar plate, and 10‍ ‍μL of the above ethyl acetate extract was dispensed on the disks. The Petri dishes were incubated at 28°C for 12 h, and the formation of violacein showing a visible purple color was examined around the disks.

The detection of AHL molecules by GC/MS was performed using a GCMS-QP5050A gas chromatograph (Shimadzu) equipped with a HP-5MS capillary column (Agilent Technologies) as previously described (Cataldi *et al.*, 2004). Briefly, 1‍ ‍μL of the ethyl acetate extract was injected into the gas chromatograph in a splitless mode. Helium gas was used as the carrier gas at a flow rate of 0.8‍ ‍mL min^–1^. The temperatures of the injector and interface were set at 200 and 280°C, respectively, and the column temperature was increased from 150 to 275°C at intervals of 15°C min^–1^. Mass spectra were obtained by the electron ionization mode and scanned from 40 to 160 *m/z*.

### Bioinformatic analysis

The presence and absence of orthologues of the known genes involved in the biosynthesis and reception of AHL molecules were examined using the *B. sinica* genome (accession number; BAFN01000000). To achieve this, a blastp search was performed with the query sequences shown in Table S1. The blastp search was performed using blast-2.2.14 software. The alignment of the protein sequence was conducted by MUSCLE 3.6 software with the default condition (16 iterations) (Edgar, 2004). The phylogenetic tree was calculated by MEGA7 using the maximum likelihood (Jones-Taylor-Thornton model) method (Kumar *et al.*, 2016).

### Transcription analysis

Total RNA extraction, cDNA synthesis, and PCR amplification for the detection of transcripts of the BROSI_A2810, BROSI_A1042, and BROSI_A3652 genes were performed as previously described (Oshiki *et al.*, 2017). Briefly, total RNA was extracted using a RNeasy Mini kit (Qiagen) according to the instruction manual supplied by the manufacturer. Contaminated DNA molecules were removed using the TURBO DNase-free kit (Thermo Fisher Scientific). The RT reaction was conducted at 42°C for 60‍ ‍min using a PrimeScript II 1^st^ strand cDNA synthesis kit (TakaraBio) and random-hexamer oligonucleotide primers. Synthesized cDNA was subjected to PCR amplification using Ex Taq polymerase (TakaraBio). The oligonucleotide primers used in the present study were as follows: BROSI_A2810-forward (5′-TGTCTCACTCTGGATGCTTTCTAC-3′), BROSI_A2810-reverse (AATCAGTGAAATACCCCTTTTGAA), BROSI_A1042-forward (GAAAGGGGATTAATCATTCAACAG), BROSI_A1042-reverse (GCCAAAGGTTACTTTAATTCTGGA), BROSI_A3652-forward (AAACCAGATATCCTCATTACTGATTTTC), and BROSI_A3652-reverse (TTATATGGAAGAGGACCAATGTCTGATAG). PCR amplicons were subjected to 1.5% agarose gel electrophoresis, which was subsequently detected by staining with the SYBR safe DNA gel stain (Life Technologies). The lengths of PCR amplicons were assessed by comparisons with molecular size markers. As negative controls, milliQ water (*i.e.*, without DNA and RNA molecules) and extracted RNA without the RT reaction were also subjected to RT-PCR. The specific amplification of genomic regions in which oligonucleotide primers were hybridized was confirmed by elucidating the nucleotide sequences of PCR amplicons using the Sanger sequencing method.

## Results

### Cell density-dependent occurrence of anammox activities

**Fig. 1.**
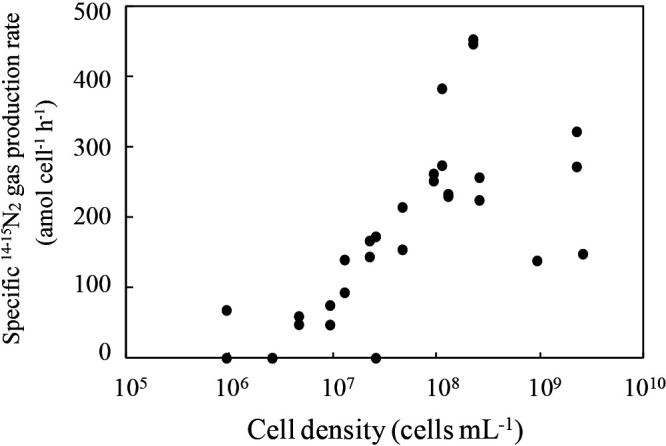
Cell density-dependent activity of ^14-15^N_2_ gas production. Planktonic *Brocadia sinica* cells were serially diluted to a cell density of 10^6^–10^10^‍ ‍cells‍ ‍mL^–1^, and anoxically incubated with the addition of 2.5‍ ‍mM ^15^NH_4_^+^ and ^14^NO_2_^–^. The production of ^14-15^N_2_ gas, which is specifically produced from the anammox process was assessed by gas chromatography mass spectrometry (GC/MS).

Planktonic cells of *B. sinica* (Fig. S1a) were serially diluted from 10^6^–10^10^‍ ‍cells‍ ‍mL^–1^, and anoxically incubated with the addition of 2.5‍ ‍mM ^15^NH_4_^+^ and ^14^NO_2_^–^. The cell-specific ^14-15^N_2_ gas production rate (defined as specific anammox activity) was measured (Fig. 1). ^14-15^N_2_ gas production was not detected in control vials without *B. sinica* cells. Specific anammox activities were greater than 150‍ ‍amol cell^–1^ h^–1^ when cell densities were set to be greater than 10^8^‍ ‍cells‍ ‍mL^–1^. Specific activity decreased with reductions in cell density and was <100 amol cell^–1^ h^–1^ or often undetectable at a cell density of <10^7^‍ ‍cells‍ ‍mL^–1^.

The anoxic incubation was repeated using the dispersed granular biomass of *B. sinica* (the diameters of the fragmented biomass ranged between 50 and 300‍ ‍μm [Fig. S1b]), and the cell-specific ^14-15^N_2_ gas production rate was assessed at different cell densities (Fig. S2). Specific anammox activity was generally lower than that of planktonic cells; *i.e.*, the highest activities obtained using the planktonic and dispersed granular biomasses were 457 and 63 amol cell^–1^ h^–1^, respectively. The specific ^14-15^N_2_ gas production rate decreased with reductions in cell density, similar to planktonic cells. This activity was undetectable when the cell density was less than 10^8^‍ ‍cells‍ ‍mL^–1^, which was one order of magnitude higher than that for planktonic cells.

Although cell-density dependent anammox activity experiments were performed using a highly enriched culture of *B. sinica*, some contaminants (*i.e.*, nitrifiers and heterotrophic denitrifiers) were present in the enrichment culture, which may have influenced the results obtained (Fig. 1 and S2). Therefore, the incubation was repeated with the addition of penicillin G to suppress the microbial activity of contaminating microorganisms, but not of anammox bacteria (van de Graaf *et al.*, 1996; Güven *et al.*, 2005). Planktonic *B. sinica* cells were anoxically incubated at cell densities of 8.7×10^6^–10^8^‍ ‍cells‍ ‍mL^–1^ with the addition of penicillin G. The addition of penicillin G increased the specific ^14-15^N_2_ gas production rate at 8.7×10^7^‍ ‍cells‍ ‍mL^–1^ (*P*<0.05) (Fig. S3); however, activity was not detectable at 8.7×10^6^‍ ‍cells‍ ‍mL^–1^ with and without penicillin G.

### Genome-wide screening of genes involved in cell density-dependent anammox activities of *B. sinica*

The presence and absence of the genes involved in the biosynthesis and reception of known QS signal molecules (Table S1) were examined by a homology search using the *B. sinica* genome. Orthologues of *Acidithiobacillus ferrooxidans hdtS* (the BROSI_A2810 and BROSI_A1042 genes) and *Pseudomonas chlororaphis phzR* (the BROSI_A3652 gene) were found (Table 1). Phylogenetic trees of the BROSI_A2810, BROSI_A1042, and BROSI_A3652 genes were constructed (Fig. S4 and S5).

**Fig. 2.**
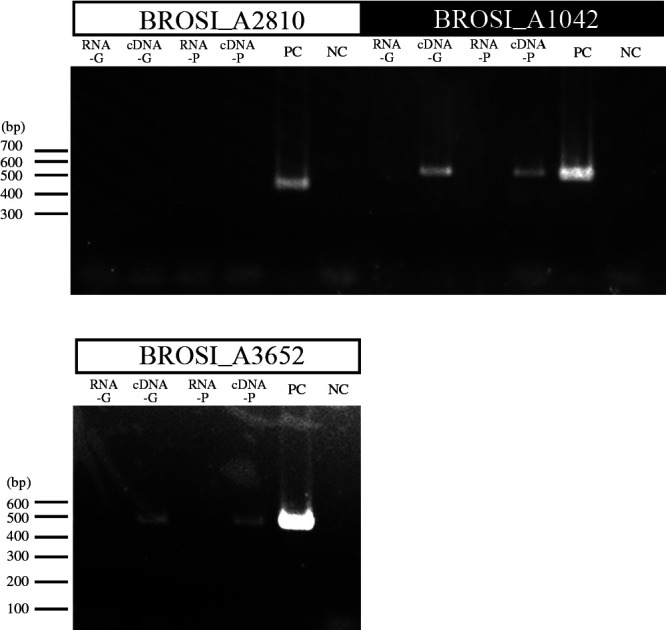
Transcription analysis of candidate genes involved in *N*-acyl homoserine lactone (AHL) biosynthesis and reception. Total RNA samples were extracted from dispersed granular and planktonic biomasses (cell density; 10^9^‍ ‍cells‍ ‍mL^–1^) of *Brocadia sinica* (designated as RNA-G and RNA-P, respectively), and subjected to cDNA synthesis (cDNA-G and cDNA-P, respectively) and subsequent RT-PCR experiments. As positive and negative controls of PCR amplification, PCR was performed using the genomic DNA of *B. sinica* and distilled water as a template (PC and NC, respectively). Locus tags of the target genes registered in the *B. sinica* genome are shown at the top of electrophoresis images. Amplicons of expected molecular sizes were detected on all gels.

An RT-PCR assay was performed to examine the transcription of the BROSI_A2810, BROSI_A1042, and BROSI_A3652 genes. Total RNAs were extracted from the dispersed granular biomass and planktonic cells (cell density; 10^9^‍ ‍cells‍ ‍mL^–1^) and used as a template of the RT-PCR assay. The specific amplification of the BROSI_A1042 (*hdtS*) and BROSI_A3652 (*phzR*) genes was confirmed from cDNA, but not from RNA samples without the RT reaction (Fig. 2), indicating that these genes were transcribed in both granular and planktonic cells. Since *hdtS* and *phzR* are known to be involved in AHL biosynthesis (Chain *et al.*, 2003; Burton *et al.*, 2005; Rivas *et al.*, 2007) and reception (Maddula *et al.*, 2006; Khan *et al.*, 2007), respectively, the transcription of the BROSI_A1042 and BROSI_A3652 genes suggests that *B. sinica* synthesized and received AHL molecules in the culture.

### Detection of AHL molecules in the supernatant of the *B. sinica* culture

The presence of AHL molecules in the culture supernatant of the granular and planktonic biomasses (*i.e.*, effluent of the up-flow column reactor and membrane filtrate of the MBR, respectively) was examined by ethyl acetate extraction followed by the AHL reporter assay and GC/MS analysis. Regarding the AHL reporter assay, the strain VIR24 produced violacein when the ethyl acetate extract of the planktonic biomass culture was spotted (Table 2). In contrast, violacein production was not observed from the ethyl acetate extract of the granular biomass culture or the inorganic medium supplied into the MBR.

**Fig. 3.**
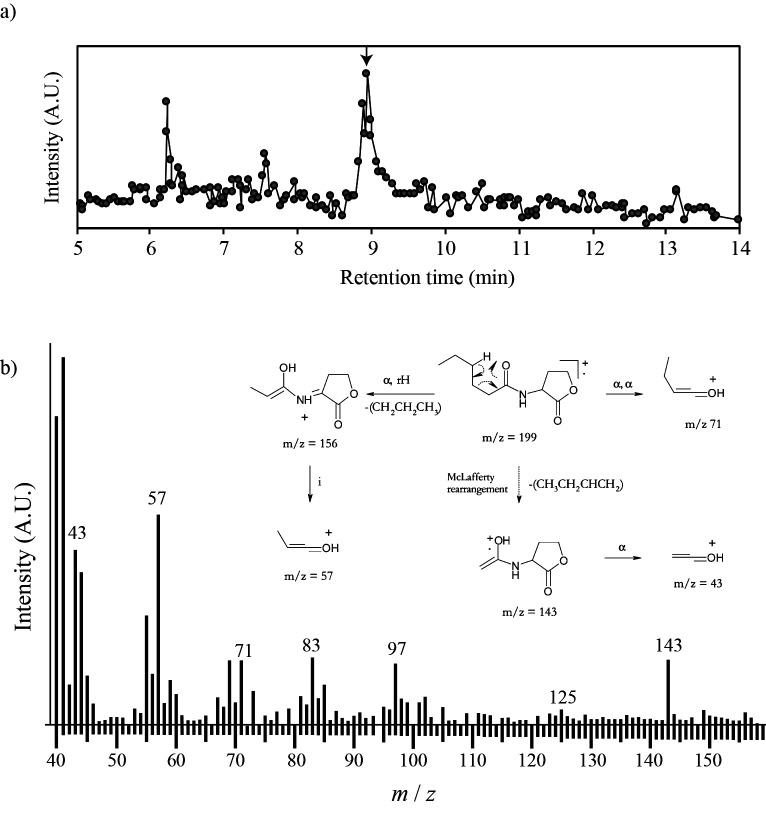
Detection of acyl homoserine lactone (AHL) from a culture of *Brocadia sinica* by gas chromatography mass spectrometry (GC/MS). AHL molecules were extracted using ethyl acetate from the effluent of a membrane bioreactor operated for the cultivation of *B. sinica*, and the extract was concentrated using a rotary evaporator. The presence of AHL molecules in the concentrated ethyl acetate extract was examined by GC/MS. Panel a) ion chromatograph at *m/z* 143, which corresponds to the *m/z* of a prominent fragmentation peak of AHL molecules. A peak appeared at a retention time of 9.24‍ ‍min, and the mass spectrum of the peak was shown in panel b. The fragmentation pattern of the AHL molecule and corresponding *m/z* was shown as a superimposed image, which was referred from the study by Cataldi *et al.* (2004).

The presence of AHL molecules in the ethyl acetate extract obtained from the culture of the planktonic biomass was further examined by GC/MS. When a signal intensity peak was detected at *m/z* 143, which corresponds to the *m/z* of a prominent fragmentation peak of AHL molecules (Cataldi *et al.*, 2004), a mass peak appeared at a retention time of 9.24‍ ‍min (Fig. 3a). The mass spectrum at this retention time showed the presence of mass peaks at *m/z* 43, 57, 71, and 125 (Fig. 3b), which may have been produced during the fragmentation and modification of AHL molecules (Cataldi *et al.*, 2004) (See the imposed panel in Fig. 3b). The GC/MS spectrum confirmed the presence of AHL molecules in the ethyl acetate extract obtained from the planktonic *B. sinica* MBR culture.

### Influence of AHL molecules on cell density-dependent ^14-15^N_2_ gas production

**Fig. 4.**
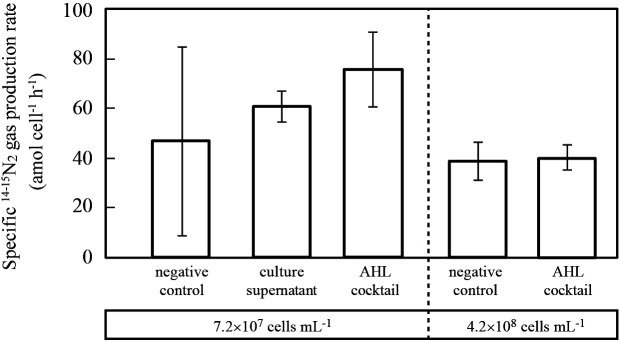
Stimulation of ^14-15^N_2_ gas production activity by the addition of concentrated ethyl acetate and a cocktail of pure acyl homoserine lactone (AHLs) (designated as the culture supernatant and AHL cocktail, respectively). Planktonic *Brocadia sinica* cells (cell density; 7.2×10^7^ and 4.2×10^8^‍ ‍cells‍ ‍mL^–1^) were anoxically incubated with the addition of 2.5‍ ‍mM ^15^NH_4_^+^ and ^14^NO_2_^–^. The error bar represents the range of the standard deviation derived from triplicate vials.

The above ethyl acetate extract of AHL containing the MBR culture and the AHL cocktail (C_6_-, C_8_-, C_10_-, and C_12_-HSL, 100‍ ‍μM) were supplemented into the planktonic *B. sinica* culture with cell densities of 7.2×10^7^‍ ‍cells‍ ‍mL^–1^, and specific ^14-15^N_2_ gas production rates were then assessed (Fig. 4 and S6). The addition of the ethyl acetate extract and AHL cocktail resulted in specific anammox activity that was 1.2- and 1.6-fold higher, respectively, than that of the negative control. When the incubation was repeated at a cell density of 4.2×10^8^‍ ‍cells‍ ‍mL^–1^, no increases were detected in specific anammox activity following the addition of the AHL cocktail.

## Discussion

In the present study, cell density-dependent anammox activity was examined using planktonic *B. sinica* cells, which enabled a more accurate assessment of this activity than that using a dispersed aggregating biomass (Strous *et al.*, 1999; Egli *et al.*, 2001; Clippeleir *et al.*, 2011). The results obtained revealed that the specific anammox activity of *B. sinica* was highly dependent on cell density. The cell density threshold above which planktonic *B. sinica* cells exhibited anammox activity was 10^7^‍ ‍cells‍ ‍mL^–1^ (Fig. 1), which was an order of magnitude lower than that using the dispersed aggregating biomass (10^8^‍ ‍cells‍ ‍mL^–1^) (Fig. S1) and markedly lower than those reported previously: 10^10-11^‍ ‍cells‍ ‍mL^–1^ for *B. anammoxidans* (Strous *et al.*, 1999) and 10^8^‍ ‍cells‍ ‍mL^–1^ for *K. stuttgartiensis* (Egli *et al.*, 2001). Differences in the cell density threshold between the planktonic and dispersed aggregating biomasses may primarily be attributed to the overestimation of cell density due to the presence of other coexisting microorganisms and/or inactive anammox bacteria in the aggregating biomass. Additionally, the specific anammox activity of planktonic cells was an order of magnitude higher than that of the dispersed biomass (Fig. 1 and S2). An aggregating biomass contains a heterogeneous population, with the inner part generally containing a metabolically dormant population due to limited diffusion (Okabe *et al.*, 1999a; 1999b). In addition to this heterogeny, the aggregating biomass is damaged during the mechanical homogenization process, which may contribute to the low specific anammox activity of the dispersed biomass. These findings highlight the importance of accurate measurements of the cell density of active anammox bacteria.

The results of the AHL reporter assay and GC/MS analysis revealed the presence of AHL molecules in the MBR culture of *B. sinica*, as reported in previous studies (Tang *et al.*, 2015; 2019). The concentrations of the detected AHL molecules from the ethyl acetate extract were estimated to be in the submillimolar concentration range based on the detection limit of the AHL reporter assay. Thus, the concentration in the MBR culture needs to be a submicromolar concentration because the concentration ratio of the ethyl acetate extract was 5,000. Although AHL was found in the MBR, it currently remains unclear whether *B. sinica* or other coexisting microorganisms produced the AHL molecules because a taxonomically wide range of Gram-negative bacteria are capable of biosynthesizing AHL (Parsek and Greenberg, 2000). Penicillin G inhibits the majority of Gram-negative bacteria, but not anammox bacteria. Cell-specific ^14-15^N_2_ gas production rate measurements with the addition of penicillin G represent anammox activity without the potential contribution of AHL production by heterotrophic denitrifiers if any. The cell-specific ^14-15^N_2_ gas production rate at 8.7×10^7^‍ ‍cells‍ ‍mL^–1^ with the addition of penicillin G was higher than that without penicillin G, implying that anammox bacteria and heterotrophic denitrifiers compete for a common substrate, such as ^15^NO_2_^–^, rather than cooperate through the AHL exchange. This suggests that AHL is produced by anammox bacteria, but has to be examined experimentally. Further research is needed to elucidate the complex microbial interactions occurring among members of the *B. sinica* culture.

In order to examine whether the above AHL molecules were synthesized and received by *B. sinica* cells, the presence and absence of known genes involved in the biosynthesis and reception of AHL molecules were investigated. The *B. sinica* genome possesses the BROSI_A1042 and BROSI_A3652 genes, which appear to be involved in AHL production and reception, respectively, and the transcription of these genes was demonstrated, as shown in Fig. 2. This transcription suggested the involvement of these genes in the AHL-dependent regulation of anammox activity; however, direct evidence is still missing. Two AHL synthases (JqsI-1 and JqsI-2) were identified in *Candidatus* Jettenia caeni, which synthesized four types of AHLs in a recombinant *E. coli* strain (Tang *et al.*, 2019). Although the involvement of the BROSI_A1042 gene in AHL biosynthesis was examined, AHL production was not detected, even after several efforts, which may have been due to the misfolding of the expressed protein and/or the requirement of other cofactors; *e.g.*, the expression vector (pUC, pET-25b, pET28-a, pRSFDuet-1, and pMAL vectors) (data not shown). Further efforts are needed to demonstrate the involvement of the BROSI_A1042 gene (and/or another gene similar to the BROSI_A2810 gene) in AHL biosynthesis. Additionally, the function of the BROSI_A3652 protein needs to be investigated in order to clarify whether this protein receives some AHL molecules.

The presence of AHL molecules in the culture of *B. sinica* prompted us to investigate the effects of the external addition of AHL molecules (the ethyl acetate extract of the MBR culture and AHL cocktail (C_6_-, C_8_-, C_10_-, and C_12_-HSL, 100‍ ‍μM) on specific anammox activity. The addition of both AHL compounds increased the specific anammox activity of *B. sinica* cells by 1.2–1.6 fold at a cell density of 7.2×10^7^‍ ‍cells‍ ‍mL^–1^ (Fig. 4). However, the results obtained were not significant because of large variations in data, particularly in negative control data. Negative control experiments were performed at the cell density threshold for *B. sinica* (10^7^‍ ‍cells‍ ‍mL^–1^) without the addition of AHL, resulting in large variations in specific ^14-15^N_2_ gas production rates (*i.e.*, 46±38 amol cell^–1^ h^–1^). When the incubation was repeated at a higher cell density (4.2×10^8^‍ ‍cells‍ ‍mL^–1^), the clear stimulatory effects of AHL compounds were not detectable; therefore, although specific ^14-15^N_2_ gas production rates largely fluctuated, these stimulatory effects needed to be examined at the cell density threshold. In addition, since planktonic anammox bacteria cells are more susceptible to oxygen and nitrite inhibition at a low cell density, difficulties are associated with stably establishing and maintaining active anammox cultures at a low density. However, similar stimulatory effects of the addition of exogenous AHL were previously reported even though anammox activities were evaluated as the rate of NH_4_^+^ and/or NO_2_^–^ consumption (Clippeleir *et al.*, 2011) and *hzsA* expression (Tang *et al.*, 2019). Therefore, anammox activity appears to be regulated by AHL-mediated QS. Further detailed studies are needed to confirm the present results and elucidate the underlying molecular mechanism(s) for the cell density-dependent regulation of anammox activity.

## Citation

Oshiki, M., Hiraizumi, H., Satoh, H., and Okabe, S. (2020) Cell Density-dependent Anammox Activity of *Candidatus* Brocadia sinica Regulated by *N*-acyl Homoserine Lactone-mediated Quorum Sensing. *Microbes Environ ***35**: ME20086.

https://doi.org/10.1264/jsme2.ME20086

## Supplementary Material

Supplementary Material

## Figures and Tables

**Table 1.** Screening of candidate genes involved in acyl homoserine lactone (AHL) biosynthesis and reception by *Brocadia sinica*.

Query	Locus tag	Product	identity	*e*-value
*Acidithiobacillus ferrooxidans hdtS*	BROSI_A2810	1-acyl-sn-glycerol-3-phosphate acyltransferase	24%	1.0×10^–18^
	BROSI_A1042	1-acyl-sn-glycerol-3-phosphate acyltransferase	24%	2.0×10^–4^
*Pseudomonas chlororaphis phzR*	BROSI_A3653	Response regulator containing a CheY-like receiver domain and HTHDNA-binding domain	26%	1.0×10^–8^

**Table 2.** Detection of acyl homoserine lactone (AHL) molecules from a culture of *Brocadia sinica* by an AHL reporter assay. AHL molecules were extracted using ethyl acetate from the effluent of a membrane bioreactor operated for the cultivation of *B. sinica*, and the extract was concentrated using a rotary evaporator. The presence of AHL molecules in the concentrated ethyl acetate extract (designated as the culture supernatant) was examined using AHL reporter strains *Chromobacterium violaceum* CV026 and VIR24, which produce violacein when the culture contains submillimolar concentrations of short- and long-acyl-chain compounds (C_4_ to C_7_ and C_6_ to C_14_ AHLs) (Someya *et al.*, 2009). As positive and negative controls of the AHL reporter assay, a cocktail of pure AHL molecules and an ethyl acetate extract prepared from freshly prepared inorganic medium were prepared and subjected to the assay. N.D.; not detected, +; weak but visible violacein production, ++; strong violacein production.

	*C. violaceum* CV026	*C. violaceum* VIR24
Culture supernatant	N.D	+
Positive control (AHL cocktail)	+	++
Negative control (media)	N.D	N.D
