# Supplementary material for: Cell Density-dependent Anammox Activity of Candidatus Brocadia sinica Regulated by N-acyl Homoserine Lactone-mediated Quorum Sensing
Source: Microbes Environ. 2020 Oct 24;35(4):ME20086. doi: 10.1264/jsme2.ME20086 (PMC7734396; doi:10.1264/jsme2.ME20086)
Supplement: Supplementary file 1 — Supplementary Material [file 35_20086_s1.pdf]

Supplemental material

Cell-density dependent anammox activity of *Candidatus* Brocadia sinica regulated by *N*-acyl homoserine lactone-mediated quorum sensing

Mamoru Oshiki, Haruna Hiraizumi, Hisashi Satoh, and Satoshi Okabe*

Division of Environmental Engineering, Faculty of Engineering, Hokkaido University,
North-13, West-8, Sapporo, Hokkaido 060-8628, Japan.

***Corresponding author:**
Satoshi OKABE (sokabe@eng.hokudai.ac.jp)
Division of Environmental Engineering, Faculty of Engineering, Hokkaido University
North-13, West-8, Sapporo, Hokkaido 060-8628, Japan.
Tel&Fax: (+81)-011-706-6266

This file contains 6 figures, and 1 table.

1 Supplementary Figure

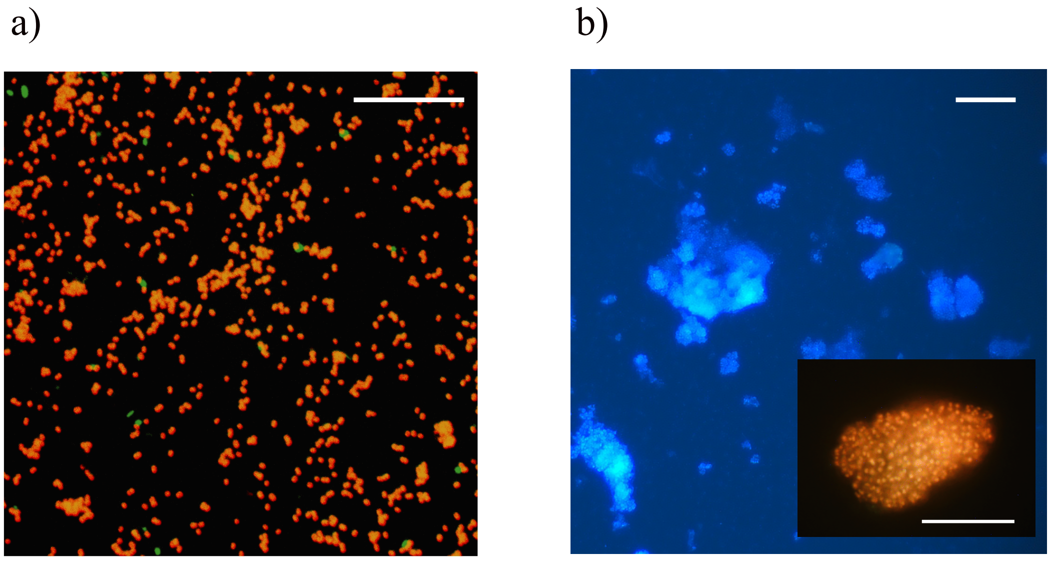

Fig. S1 (Oshiki et al.)

Fig. S1. Microscopic examination of planktonic (panel a) and dispersed *B. sinica* (panel b) biomass. a) Planktonic cells were collected from a membrane bioreactor, and hybridized with both TRITC-labeled amx820 (red) and FITC-labeled EUB mix (green) oligonucleotide probes for all the anammox bacteria and most members of the eubacteria, respectively. Scale bar = 25 µm. b) Granular biomass was dispersed into small aggregated biomass using a glass tissue homogenizer, and the cells were stained with 2-(4-amidinophenyl)-1H -indole-6-carboxamidine (DAPI). As shown in an imposed image, the small aggregated biomass was mainly composed of the microcolony of *B. sinica* as examined by fluorescence *in-situ* hybridization using the amx820 and EUB mix oligonucleotide probes. Scale bar = 100 and 25 µm for the main image and imposed image, respectively.

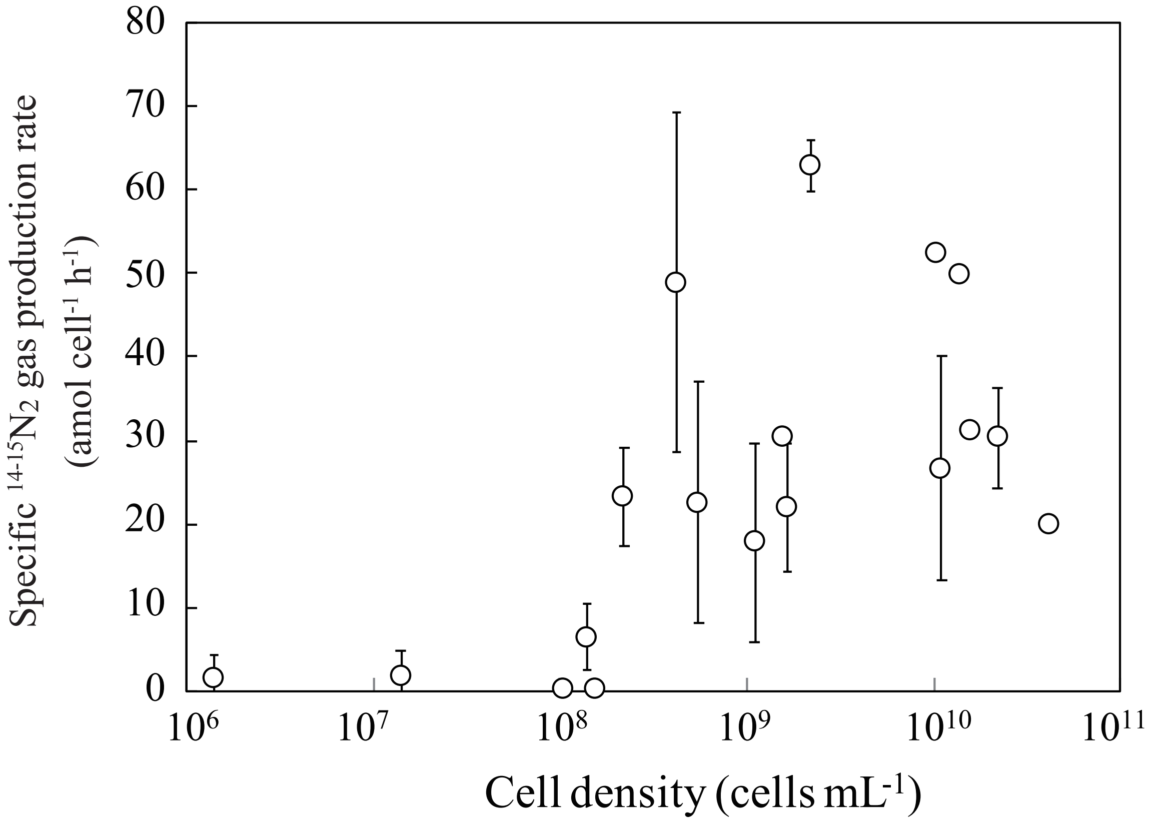

Fig. S2 (Oshiki et al.)

Fig. S2. Cell-density dependent activity of $^{14\text{-}15}N_2$ gas production examined using a dispersed granular biomass. Granular biomass of *B. sinica* was dispersed using a glass tissue homogenizer, and serially diluted to be cell density of 10^6 – 10^{11} cells mL^{-1}. The culture was anoxically incubated with addition of 2.5 mM $^{15}NH_4^+$ and $^{14}NO_2^-$. Production of $^{14\text{-}15}N_2$ gas which is specifically produced from the anammox process was examined by gas chromatography mass spectrometry (GC/MS). Error bar represents the range of standard deviation derived from triplicate vials.

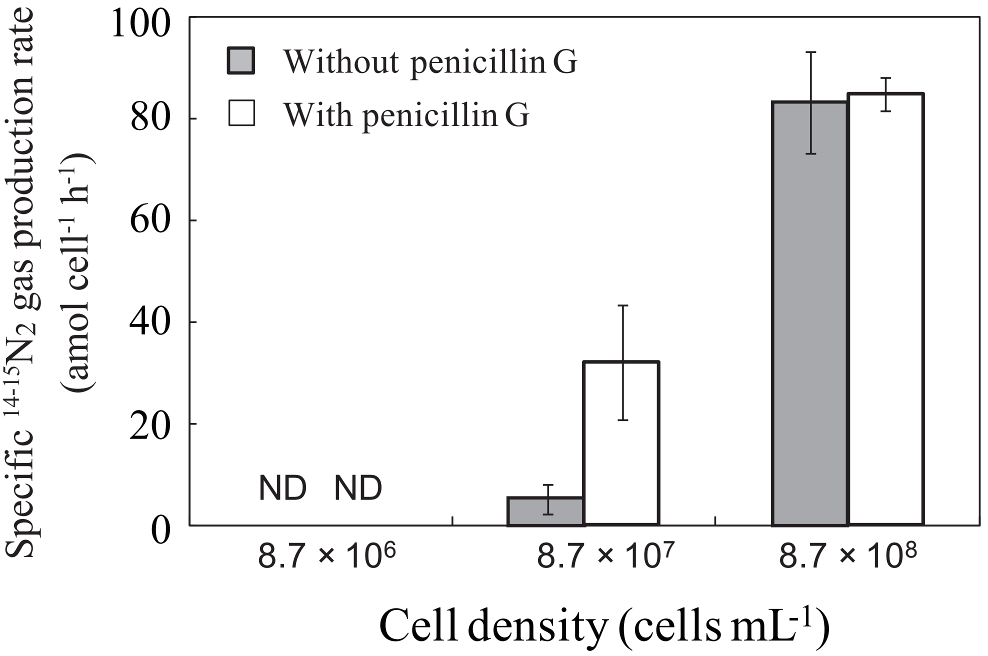

Fig. S3 (Oshiki et al.)

Fig. S3. Influence of penicillin G addition to $^{14\text{-}15}N_2$ gas production rate of *B. sinica*. Planktonic *B. sinica* cells were serially diluted to be cell density of 8.7 × 10^6 – 10^8 cells mL^{-1}, and anoxically incubated with addition of 2.5 mM $^{15}NH_4^+$ and $^{14}NO_2^-$. Penicillin G was added at the final concentration of 500 μg mL^{-1} to inhibit activity of heterotrophic denitrifier. Error bar represents the range of standard deviation derived from triplicate vials. ND; not detected.

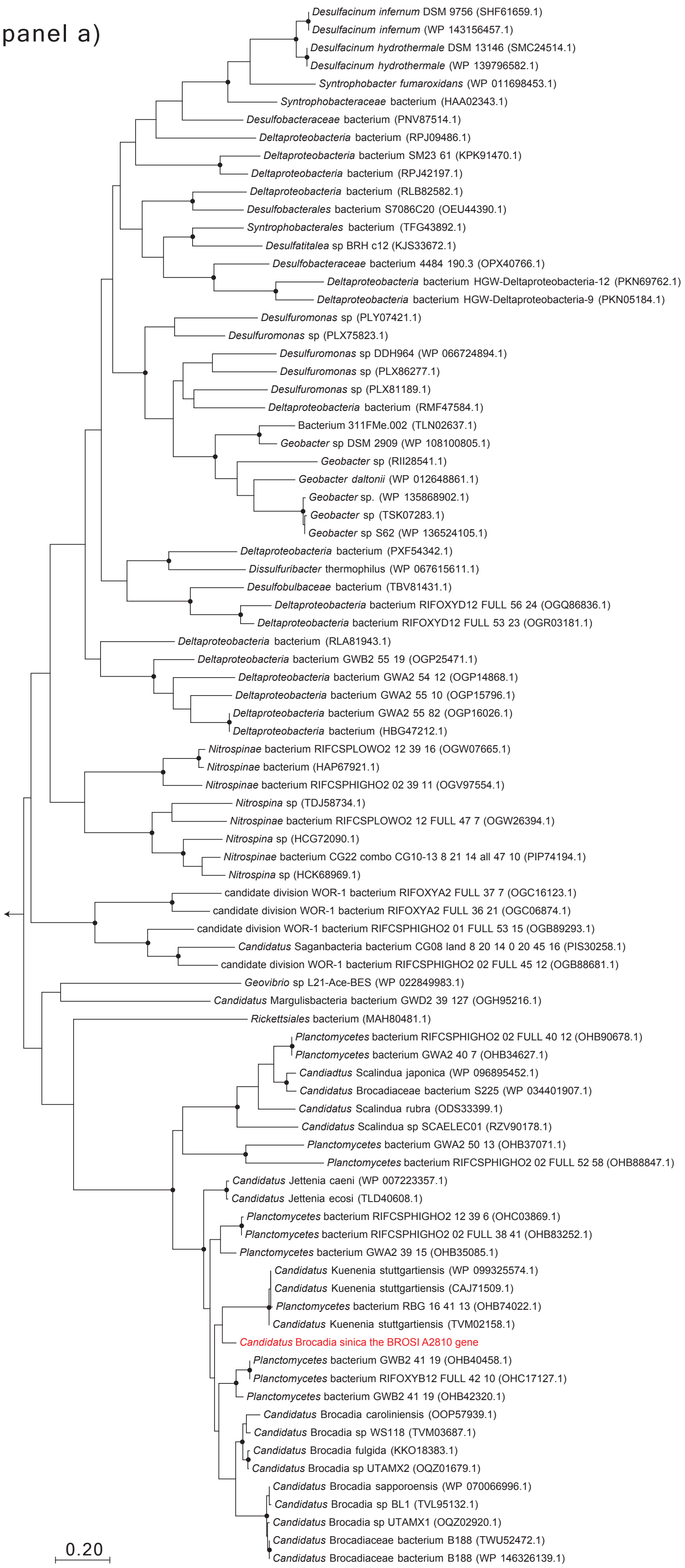

Fig. S4a (Oshiki et al.)

45

panel b)

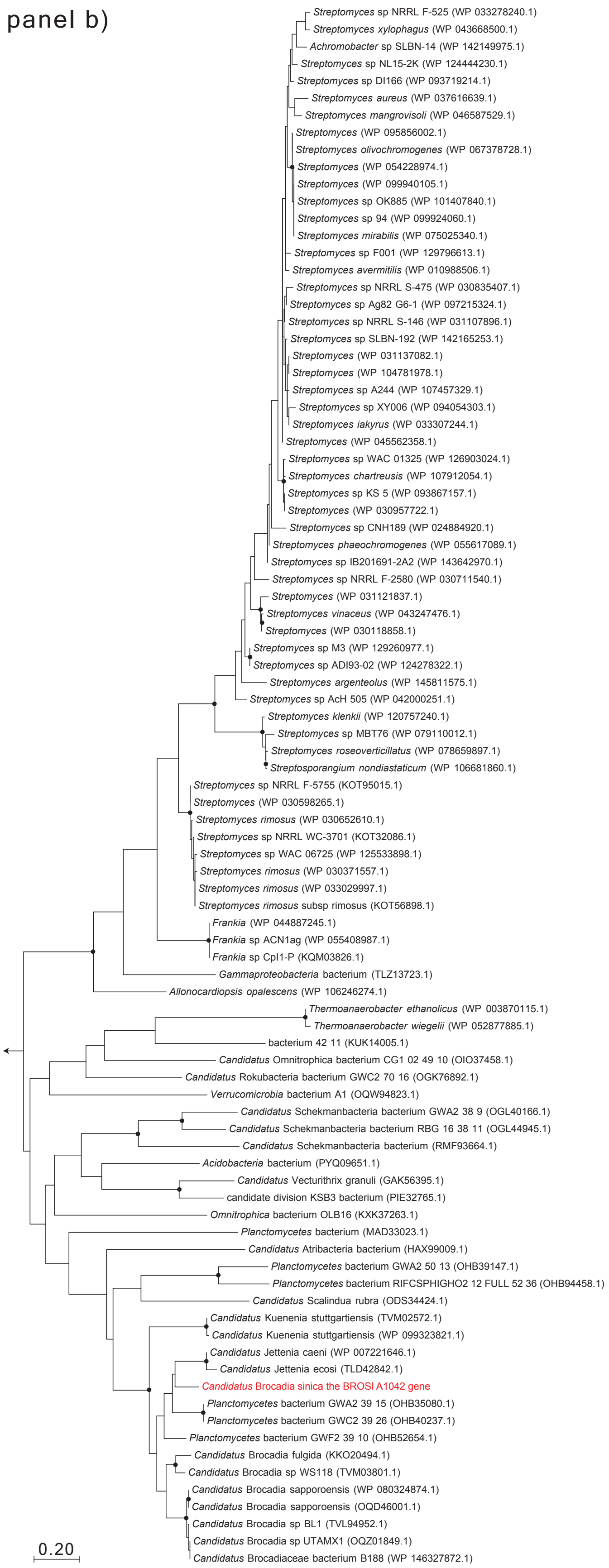

Fig. S4b (Oshiki et al.)

Fig. 4. Maximum likelihood (ML) tree showing phylogenetic affiliation of the BROSI_A2810 (panel a) and BROSI_A1042 (b) genes. Branching points that support probability >80% in the bootstrap analyses (based on 500 replicates) are shown as black circles. The scale bar represents 20% sequence divergence. The phylogenetic position of the BROSI_A2810 and BROSI_A1042 genes were shown with red color.

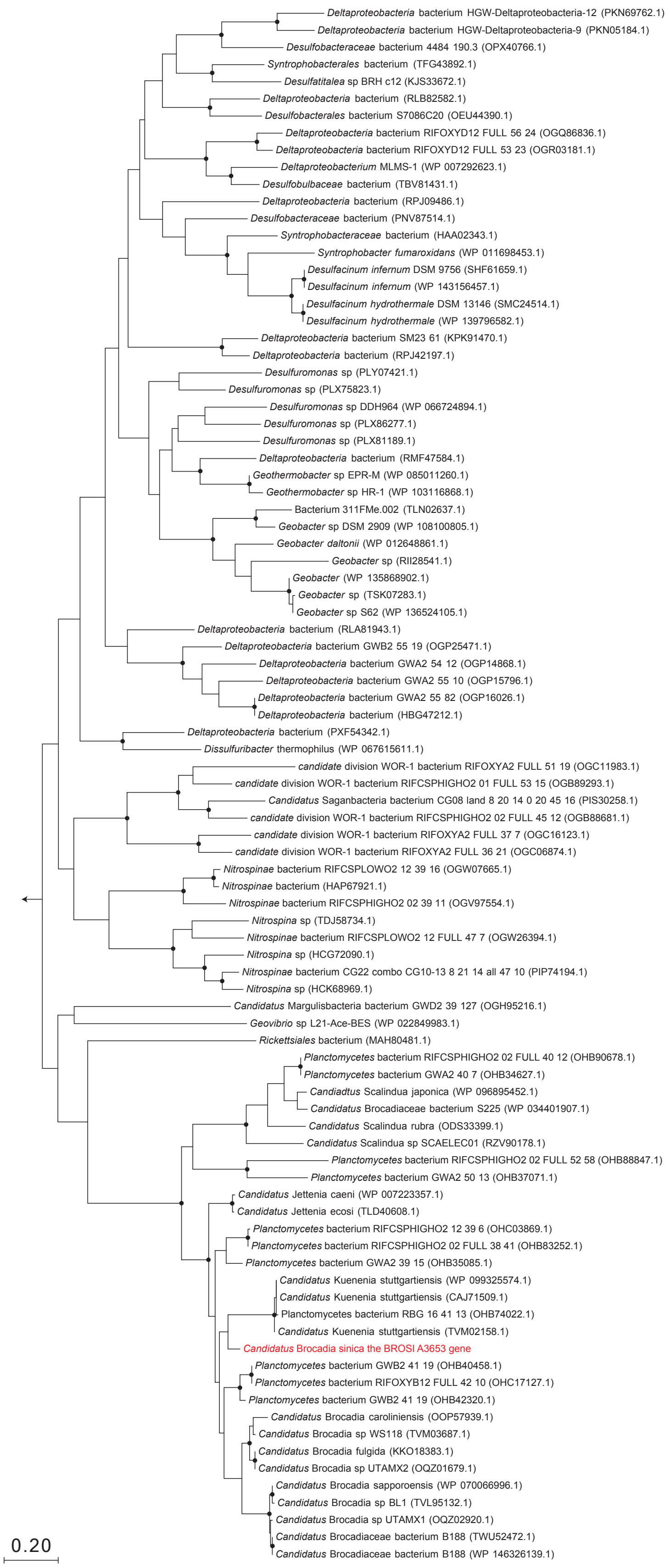

Fig. S5 (Oshiki et al.)

Fig. S5. Maximum likelihood (ML) tree showing phylogenetic affiliation of the BROSI_A3653 genes. Branching points that support probability >80% in the bootstrap analyses (based on 500 replicates) are shown as black circles. The scale bar represents 20% sequence divergence. The phylogenetic position of the BROSI_A3653 gene was shown with red color.

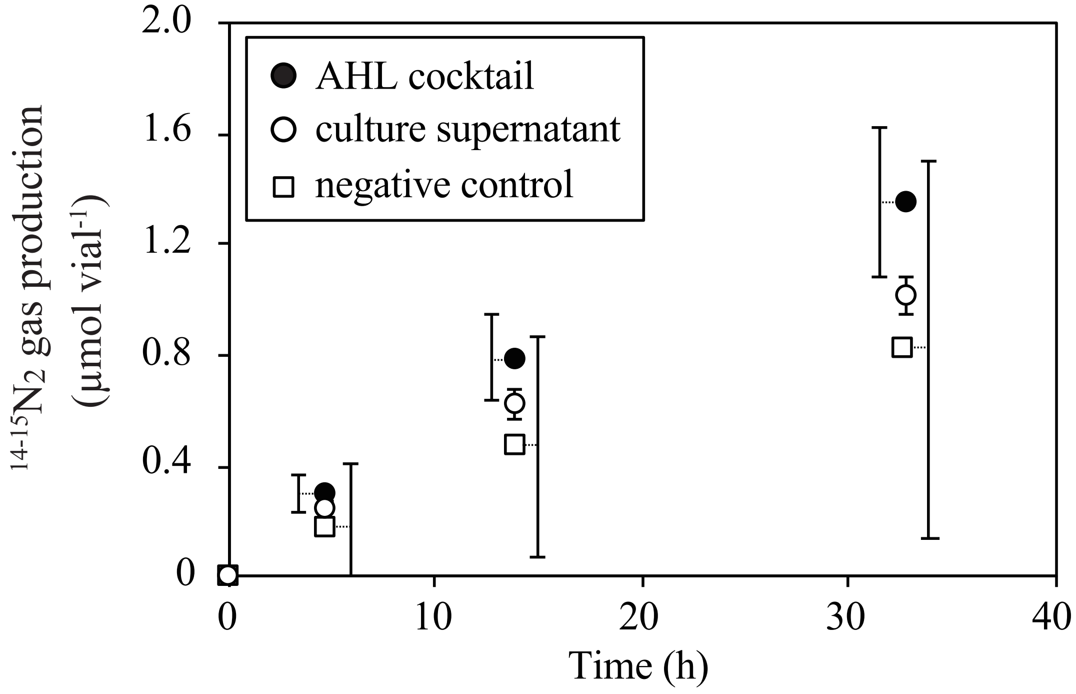

Fig. S6. Stimulation of $^{14\text{-}15}N_2$ production by planktonic *B. sinica* cells (cell density of 7.2×10^7 cells mL^{-1}) by adding the concentrated ethyl acetate and a cocktail of pure acyl homoserine lactone (AHLs) (designated as culture supernatant and AHL cocktail, respectively). Cell-specific $^{14\text{-}15}N_2$ production rates were shown in **Fig. 4**. Error bars represent the range of the standard deviation derived from triplicate incubations. As for the error bars for the plots of AHL cocktail and negative control, those were shown on the left and right side of the plots, respectively, to avoid overlap among the plots.

2. Supplementary Table

Table S1 Blast queries used for screening the genes responsible for quorum sensing of *B. sinica*.

acyl-homoserine lactone	gene	GenBank accession number
Acidithiobacillus ferrooxidans	*hdtS*	AAZ78229
Aeromonas hydrophila	*ahyR, ahyI*	X89469
Aeromonas salmonicida	*asaR, asaI*	U65741
Agrobacterium tumefaciens	*traR, traI*	L17024, L22207
Burkholderia cepacia	*cepR, cepI*	AF330018, AF330012
Enterobacter agglomerans	*eagR, eagI*	X74300
Erwinia carotovora subsp. *carotovora*	*carR, expR* *expI (carI)*	X74299, X80475, X72891
Erwinia chrysanthemi	*expR, expI (echR, echI)*	X96440
Pseudomonas aeruginosa	*lasR, lasI*	M59425
	rhlR, rhlI (vsmR, vsmI)	L08962, U11811, U15644
Pseudomonas aureofaciens	*phzR, phzI*	L32729, L33724
Ralstonia solanacearum	*solR, solI*	AF021840
Rhizobium leguminosarum	*rhiR*	M98835
Rhodobacter sphaeroides	*cerR, cerI*	AF016298
Serratia liquefaciens	*swrR, swrI*	U22823
Vibrio anguillarum	*vanR, vanI*	U69677
Vibrio fischeri	*luxR, luxI*	M19039, M96844, M25752
Yersinia enterocolitica	*yenR, yenI*	X76082
Yersinia pseudotuberculosis	*ypsR, ypsI*	AF079973
	ytbR, ytbI	AF079136
**AI-2**		
Aliivibrio logei	*luxS*	HQ400993
Erwinia carotovora	*luxS*	AJ628151
Escherichia coli	*luxS*	AJ786260
Photorhabdus luminescens	*luxS*	AJ457090
Prevotella ruminicola	*luxS*	AB094409
Ruminococcus flavefaciens	*luxS*	AB094408
Serratia marcescens	*luxS*	AJ628150
Serratia sp.	*luxS*	AJ628152
Vibrio harveyi	*luxS*	HQ401005
**A-facter(γ-butyrolactones)**		
Streptomyces griseus	*afsA, arpA*	M24250, X79605, AB462507, AB021882
**Oligopeptide**		
Bacillus subtilis	*comP, comA*	M22856
Bacillus subtilis	*comX, comQ, degQ*	AB010576
Bacillus subtilis		DQ241783
Lactococcus lactis	*nisR, nisK*	Z22813
Staphylococcus aureus	*agrD, agrC, agrB*	AJ617723
Streptococcus pneumoniae	*comC, comD, comE*	AJ240790
